# COVID-19: information access, trust and adherence to health advice among migrants in Norway

**DOI:** 10.1186/s13690-021-00764-4

**Published:** 2022-01-04

**Authors:** Ahmed A. Madar, Pierina Benavente, Elżbieta Czapka, Raquel Herrero-Arias, Jasmin Haj-Younes, Wegdan Hasha, George Deeb, Kathy A. Møen, Gaby Ortiz-Barreda, Esperanza Diaz

**Affiliations:** 1 Department of Community Medicine and Global Health, Institute of Health and Society, University of Oslo, Oslo, Norway; 2 Department of Global Public Health and Primary Care, University of Bergen, Bergen, Norway; 3 Sociology Institute, Faculty of Social Sciences, University of Gdańsk, Gdańsk, Poland; 4 Department of Health Promotion and Development, University of Bergen, Bergen, Norway; 5 Department of Pharmacy, University of Oslo, Oslo, Norway; 6 NORCE Research Centre, Bergen, Norway; 7 Research group of Public Health, University of Alicante, Alicante, Spain; 8 Unit for Migration and Health, Norwegian Institute of Public Health, Oslo, Norway

**Keywords:** COVID-19, Migrants, Preventive measures, Trust and adherence to health advice, Norway

## Abstract

**Background:**

Migrants in Norway bear a higher burden of COVID-19 infections and hospitalization as compared to non-migrants. The aim of our study was to understand how migrants perceive their own health risk, how they access information regarding the preventive measures, the degree of trust in this information, in the Norwegian authorities and the news media, and migrants’ adherence to authorities’ recommendations regarding the pandemic.

**Methods:**

An online survey was performed between May and July 2020 among 529 Polish, Arabic, Somali, Tamil, and Spanish-speaking migrants in Norway. For each outcome presented in the aims, unweighted and weighted descriptive analyses were performed for all migrants together and for each language group.

**Results:**

Sixty-one percent of migrants perceived their health as excellent or very good, with the lowest value (42%) in the Tamil group and the highest among Somalians (85%). The majority of respondents (82%) felt they had received sufficient information. Press conferences from the government, health authorities’ websites, and Norwegian news media were the preferred channels of information for all groups. Most migrants reported a high level of adherence to preventive measures (88%) and trust in Norwegian authorities (79%). However, there were variations among groups regarding the importance of sources of information and level of trust, which was lowest for the Polish group.

**Conclusion:**

Migrants in Norway reported receiving sufficient information about COVID-19 and high adherence to preventive measures. However, the levels of trust in the information sources, the services and the authorities varied among the groups. Understanding how migrants are dealing with this pandemic is crucial to improve the dissemination of information and trust in the health authorities for the different groups.

## Background

The COVID-19 pandemic has become one of the most important public health crisis in Europe and put a great toll on health and health systems worldwide. The impact on individuals and societies has been very severe, not only on health but also at the socioeconomic level, magnifying pre-existing health inequities [1, 2]. Over and above the overrepresentation of COVID-19 infected among people in the lowest socioeconomic levels, several European countries report an excessive burden of infection and higher hospitalization rates among migrants as compared to the majority populations [3–6].

In 2019, the estimation of international migrants in Europe was 82 million, which constitutes 11% of the European population [7]. According to Statistics Norway (SSB), in 2020, about 18% of the total population in Norway had migrant background (14.7% had migrated themselves and 3.5% were Norwegian born to migrant parents). Migrants in Norway come originally from 221 different countries, have different lengths of stay in the host country and represent a vast heterogeneity in terms of cultural and socioeconomic background [8].

Since March 12th of 2020, Norway has adopted different measures to prevent and delay the spread of COVID-19 [9]. Initially, the measures were aimed at the entire population and information was disseminated countrywide through several channels without specific interventions for subgroups of the population. Following international news regarding growing concern that migrants were missing important information [10], the recommendations were later translated into several languages and disseminated through a broader range of channels to reach different migrant groups. Despite this, many migrant groups in Norway seemed to have higher infection rates [11]. This was confirmed by the first official status report on COVID-19 by the Norwegian Institute of Public Health (FHI) that indicated that 21% of those infected by the virus were born outside of Norway [12]. In subsequent reports, the proportion rose to 31% among the infected and 36% among the hospitalized. According to the weekly reports from the Norwegian Public health Institute, persons with Somali background had the highest proportion (570/5089) in the infected group among immigrants since the beginning of the pandemic, while the number of infected Polish migrants increased to 518 cases by October 2020 [13].

It is expected that some recommendations will be maintained for several months, perhaps one to two years. COVID-19 cannot be controlled if some groups of the population are left behind without adequate information about the containment strategies. Dissemination of reliable and clear information in an appropriate language is essential to obtain long-term adherence to the recommendations in all segments of society. Information on prevention and control of the spread of COVID-19 in Norway was translated some weeks after the information in Norwegian was released. However, it is still unknown if the translated information reaches migrants fast enough, if it is clear enough, if it is trusted and to which extent different migrant groups adhere to these recommendations.

In an attempt to give answers to these questions, our research group initiated the project Inncovid. Norge, which included a nationwide online survey among migrants in Norway with mother tongue Polish, Arabic, Somali, Tamil and Spanish. The study aimed to describe how migrants perceived their situation during the first wave of the COVID-19 pandemic. Specifically, we aimed to understand how migrants perceived their own health risk, how they accessed information regarding the pandemic and the preventive measures recommended by the health authorities, the degree of trust in this information, in the health authorities, the government and the Norwegian news media, and migrants’ adherence to the recommendations. This information will be of strategic value to advise the health and political authorities so that they can adapt and disseminate information and recommendations about the corona pandemic through proper, trustworthy and relevant channels.

## Methods

### Survey

As a part of the project Inncovid. Norge [14], an online survey was developed based on the Norwegian Citizen Panel (NCP) survey performed in March of 2020 in relation to the pandemic. The Inncovid. Norge survey included 45 questions about the participant’s and his/her family’s general health, risk assessment, sources of information about the outbreak, how they were affected by the pandemic, knowledge about and adherence to government recommendations, and the degree of trust they had in several Norwegian institutions to handle the pandemic. In addition, the survey contained eight socio-demographic questions. The initial questionnaire was developed in Norwegian and then translated into five different languages: Polish, Arabic, Somali, Tamil and Spanish. Two bilingual members of the project team performed translations for each language. We used web-based software (Nettskjema) to create and run the survey. This portal collects data directly into a secure server (TSD). All participants were registered with a serial number and no personally identifiable information was recorded. Data was collected between May 25th and July 1st of 2020. All participants received information about the study and provided consent before responding. The survey’s completion time was around 15 min.

### Respondents

We recruited migrants living in Norway who could complete the surveys in Polish, Arabic (mainly targeting Syrians), Somali, Tamil (mostly targeting people from Sri Lanka) and Spanish (primarily targeting migrants from Chile and Spain). These migrant groups were chosen as they cover different continents and the major religions in Norway, have different length of stay in Norway, reasons for migration and integration profiles. The largest group of migrants in Norway is comprised of more than 100,000 migrant workers from Poland. Migrants from Syria have had the strongest growth in Norway in recent years and, together with persons with Somali and Sri Lankan background, represent some of the biggest groups that arrived in Norway as asylum seekers/refugees. Spanish-speaking migrants constitute an important but heterogeneous migrant group in Norway, including Latin Americans and Spanish people [8]. As numbers of migrants are usually underrepresented in population surveys, we decided to obtain at least a similar proportion of migrants as the proportion of the majority population represented in the NCP. Thus, our goal was to include at least 500 of the approximately 200,000 migrants from the mentioned groups living in Norway (0.25%) to achieve a similar proportion of the population as the NCP survey, which was responded to by 12,051 individuals among 5.4 million persons (0.22%).

Project researchers, whose mother tongue is one of these five languages, reached the target groups through key members of the migrant communities, informal networks using a snowball sampling method. We encouraged migrants to further forward invitations -using a survey link- through Messenger, Viber, WhatsApp and other social media. Information about the project was also forecasted in local radio channels and posted on relevant Facebook groups used by migrants living in Norway and on the websites of the Norwegian Organization for Asylum Seekers (NOAS), Moja Norwegia (portal for Polish migrants in Norway) and Church City Mission.

### Variables and data analysis

This study reports the answers to 31 variables, eight of these were demographic. For reporting purposes and facilitating comparisons between groups, Likert scales from 16 variables were merged:

- 5-point Likert scales were merged into 3-point Likert scales (for variables “My health is”, “My infection risk in 2020 is”, “Extent of trust in Norwegian news media”, “I worry the COVID-19 information I receive from social media is inaccurate”, “Level of trust I have in health authorities handling the pandemic in a good way”, “Extent of trust in Norwegian news media”, “I have followed the authorities’ advice”, and “Most Norwegians have followed the authorities’ advice”,).
- 6-point Likert scales were merged into 4-point Likert scales (for variables “Importance of press conferences from the government”, “Importance of Norwegian news media coverage”, “Importance of the information shared on social media”, “Importance of the conversations with friends and acquaintances”, “Importance of social media post from family, friends and acquaintances”, “Importance of the health authorities’ websites” and “Importance of information from my workplace/place of study”).
- 7-point Likert scales were merged into 3-point Likert scales (for variables “Level of confidence I have on receiving good medical treatment if I become seriously ill”, “The government treats all groups fairly” and “The government listens to the opinions of all the citizens”).
- 8-point Likert scales were merged into 3-point Likert scales (for variable “Level of trust I have in the health system (in general)”).

The analyses were conducted for all migrants together and in five separate language groups. We used R 3.4.4. for the statistical analysis. Unweighted descriptive analysis was used for demographics, COVID-19 cases and perception of health. We weighted responses by gender and age and assigned an adjustment weight to each survey respondent for questions about following authorities’ recommendations, source of information and trust [15]. The weights were calculated based on a known age-gender distribution in the Norwegian population obtained from the National Statistical Institute of Norway [16]. The “survey” package in R was used for the unweighted and weighted descriptive analysis [17].

### Ethics

The Inncovid. Norge project received ethical approval from the Regional Ethical Committee (REK number 132585). The participants signed a digital consent written in their native tongue before starting to fill the questionnaires.

## Results

A total of 529 migrants responded to the survey in the five languages: 174 Polish (32.9%), 137 Arabic (25.9%), 113 Spanish (21.4%), 72 Tamil (13.6%) and 33 in Somali (6.2%%). Table 1 shows the demographic data for all migrants together and by language group. Fifty-two percent of the respondents were men and 81% were between 26 and 55 years of age. There were differences among groups in all regards except for the high percentage of persons with paid job before the pandemic in all groups. The majority of respondents (64.6%) in the Spanish group were women and 72.2% of Tamil speaking respondents were older than 45 years. Moreover, most Arabs (56.2%) arrived in Norway only 3–5 years ago, while nearly all Tamils (98.6%) had been living in this country for more than five years.

**Table 1:** Demographics, COVID-19 cases and perception of health (unweighted absolute and relative frequencies)

Variables	ALL n (%) (***N*** = 529)	Somali n (%) (***N*** = 33)	Arabic n (%) (***N*** = 137)	Tamil n (%) (***N*** = 72)	Spanish n (%) (***N*** = 113)	Polish n (%) (***N*** = 174)
**Sex**						
Female	252 (47.6)	13 (39.4)	56 (40.9)	37 (51.4)	73 (64.6)	73 (42.0)
Male	277 (52.4)	20 (60.6)	81 (59.1)	35 (48.6)	40 (35.4)	101 (58.1)
**Age**						
18–25	44 (8.3)	1 (3.0)	24 (17.5)	3 (4.2)	11 (9.7)	5 (2.9)
26–35	159 (30.1)	6 (18.2)	59 (43.1)	6 (8.3)	39 (34.5)	49 (28.2)
36–45	148 (28.0)	7 (21.2)	36 (26.3)	11 (15.3)	39 (34.5)	55 (31.6)
46–55	123 (23.3)	14 (42.4)	15 (11.0)	33 (45.8)	17 (15.0)	44 (25.3)
56–65	46 (8.7)	4 (12.1)	2 (1.5)	17 (23.6)	4 (3.5)	19 (10.9)
66+	9 (1.7)	1 (3.0)	1 (0.7)	2 (2.8)	3 (2.7)	2 (1.2)
**Years in Norway**						
0–2	58 (11.0)	4 (12.1)	19 (13.9)	0 (0.0)	19 (16.8)	16 (9.2)
3–5	115 (21.7)	1 (3.0)	77 (56.2)	1 (1.4)	16 (14.2)	20 (11.5)
5+	356 (67.3)	28 (84.9)	41 (29.9)	71 (98.6)	78 (69.0)	138 (79.3)
**Number of children**						
0	194 (36.7)	12 (36.4)	62 (45.3)	10 (13.9)	57 (50.4)	53 (30.5)
1–2	231 (43.7)	12 (36.4)	37 (27.0)	35 (48.6)	46 (40.7)	101 (58.1)
3+	104 (19.6)	9 (27.2)	38 (27.7)	27 (37.5)	10 (8.9)	20 (11.4)
**People I live with**						
alone	97 (18.3)	4 (12.1)	32 (23.4)	7 (9.7)	13 (11.5)	41 (23.6)
family members	368 (69.6)	26 (78.8)	96 (70.1)	64 (88.9)	80 (70.8)	102 (58.6)
others	64 (12.1)	3 (9.1)	9 (6.6)	1 (1.4)	20 (17.7)	31 (17.8)
**Number of people I live with**						
1–2	145 (33.6)	8 (27.6)	26 (24.8)	8 (12.3)	46 (46.0)	57 (42.9)
3–4	197 (45.6)	10 (34.5)	48 (45.7)	36 (55.4)	45 (45.0)	58 (43.6)
5+	90 (20.8)	11 (37.9)	31 (29.5)	21 (32.3)	9 (9.0)	18 (13.5)
**Paid job before Covid-19 pandemic**						
Yes	441 (83.4)	27 (81.8)	105 (76.6)	64 (88.9)	95 (84.1)	150 (86.2)
**I have/had COVID-19**						
Yes, doc/test	5 (1.0)	1 (3.0)	1 (0.7)	1 (1.4)	1 (0.9)	1 (0.6)
Yes, presume	14 (2.7)	2 (6.1)	3 (2.2)	2 (2.8)	2 (1.8)	5 (2.9)
No, doc/test	40 (7.6)	2 (6.0)	10 (7.3)	13 (18.1)	4 (3.5)	11 (6.3)
No, presume	470 (88.9)	28 (84.9)	123 (89.8)	56 (77.8)	106 (93.8)	157 (90.2)
**My health is**						
Excellent or very good	321 (60.7)	28 (84.9)	84 (61.3)	30 (41.7)	76 (67.3)	103 (59.2)
Good	171 (32.3)	4 (12.1)	45 (32.9)	30 (41.7)	32 (28.3)	60 (34.5)
Fairly good or bad	37 (7.0)	1 (3.0)	8 (5.8)	12 (16.7)	5 (4.4)	11 (6.3)
**I consider myself to belong to a group that is vulnerable to the coronavirus**						
Yes	101 (19.1)	5 (15.2)	24 (17.5)	22 (30.6)	21 (18.6)	29 (16.7)
**I live with someone who belongs to a group that is vulnerable to the coronavirus**						
Yes	89 (16.8)	4 (12.1)	21 (15.3)	18 (25.0)	13 (11.5)	33 (19.0)
**My infection risk in 2020 is**						
Very or somewhat high	69 (13.1)	3 (9.1)	11 (8.0)	16 (22.2)	15 (13.3)	24 (13.8)
Middle	222 (42.0)	16 (48.5)	66 (48.2)	30 (41.7)	55 (48.7)	55 (31.6)
Somewhat or very low	238 (45.0)	14 (42.4)	60 (43.8)	26 (36.1)	43 (38.1)	95 (54.6)
**I feel I have received sufficient information from the health authorities**						
Yes	434 (82.0)	31 (93.9)	113 (82.5)	63 (87.5)	95 (84.1)	132 (75.9)
**I worry the COVID-19 information I receive from social media is inaccurate**						
Agree	416 (78.6)	20 (60.6)	90 (65.7)	53 (73.6)	93 (82.3)	160 (92.0)
Neither agree nor disagree	66 (12.5)	3 (9.1)	29 (21.2)	13 (18.1)	14 (12.4)	7 (4.0)
Disagree	47 (8.9)	10 (30.3)	18 (13.1)	6 (8.3)	6 (5.3)	7 (4.0)

### COVID-19 self-reported cases and perception of health

Self-reported COVID-19 cases were similarly distributed among all groups. In total, 96.5% of the respondents presumed or confirmed not having COVID-19. There was otherwise variation in the perception of health and risk of infection. Health was perceived as “excellent or very good” twice as often in the Somalian group compared to the Tamil one. In addition, Tamils perceived that their infection risk in 2020 was “very or somewhat high” more often than the other groups. (Table 1).

### Importance of information sources about coronavirus

**Fig. 1:**
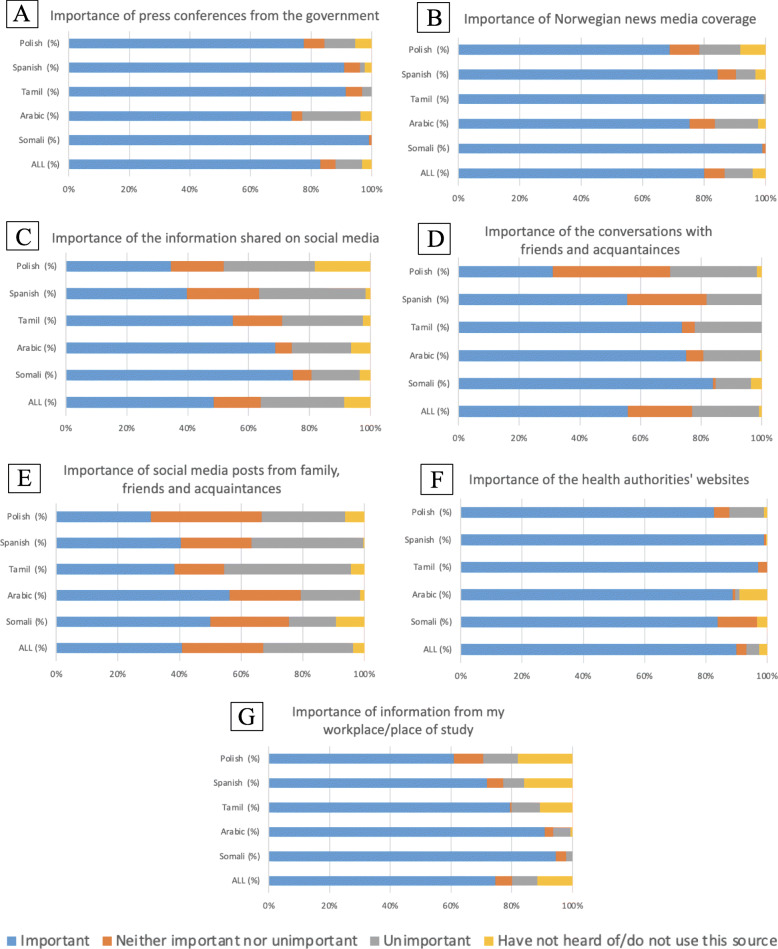
Importance of information sources (weighted values)

The vast majority of respondents (82%) felt they had received sufficient information about the coronavirus from the health authorities (Table 1). Figure 1 shows the level of importance of seven different information sources. In general, press conferences from the government (Fig. 1A), Norwegian news media (Fig. 1B) and health authorities’ websites (Fig. 1F) were relevant sources of information for all groups. Information from social media (Fig. 1C and D) and conversations with friends and acquaintances (Fig. 1E) were less important, although there were differences among groups. The Somalian group considered information obtained via social media and conversations with friends more important than other groups. In contrast, the Poles considered these sources of information less relevant than the other groups and were more worried about their inaccuracy (Table 1).

### Trust in the health system, government and Norwegian news media

**Fig. 2:**
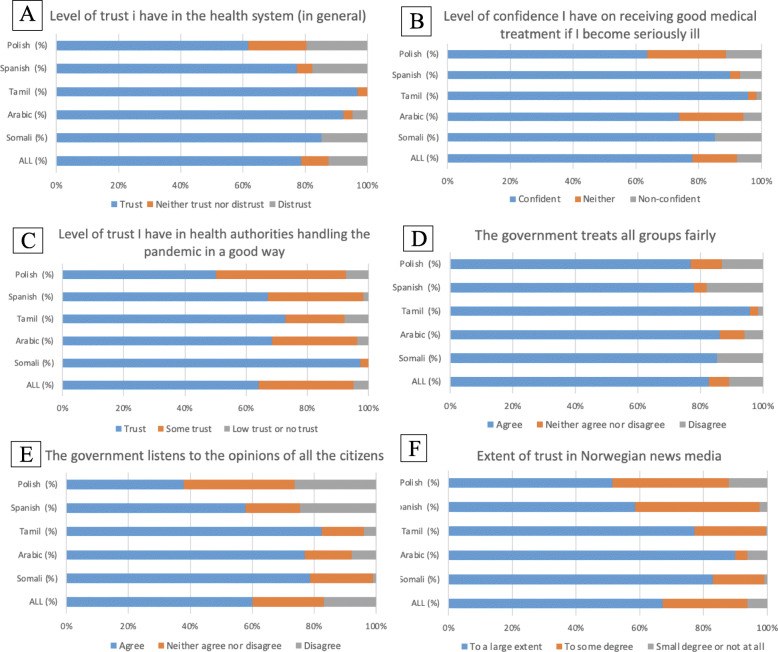
Trust in the health system, government, and Norwegian news media (weighted values)

Figure 2 shows the answers to five questions related to the extent of trust of the respondents. Overall, most participants reported trusting the health system (in general terms) (Fig. 2A and B). However, they reported less trust in how the health authorities have handled the pandemic (Fig. 2C). The majority agreed that the government treats all groups fairly (Fig. 2D). To a lesser extent, participants agreed when asked if the government listens to the opinions of all citizens (Fig. 2E). In addition, there were variations among the groups for these questions, with Poles indicating generally lower levels of trust and Tamil and Arabic speaking respondents reporting high trust in the health system. However, Arabic speaking respondents were less confident on receiving good medical treatment if becoming seriously ill.

### Following authorities’ advice

**Fig. 3:**
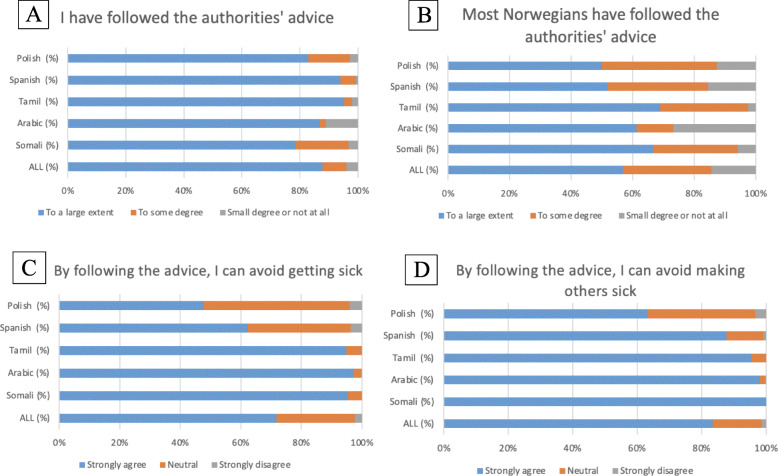
Following authorities’ advice (weighted values)

Figure 3 displays the answers to four survey questions related to following authorities’ advice. Overall, all groups reported a high level of adherence to the authorities’ advice (Fig. 3A) but perceived that Norwegians’ followed recommendations to a lesser extent (Fig. 3B). Although the majority agreed that by following the recommendations, they avoid becoming sick, Spanish and Polish speaking were more skeptical (62 and 48% for Spanish and Polish groups respectively “strongly agreed” that by following recommendations they avoid getting sick) as shown in Fig. 3C. The Polish group also reported skepticism about avoiding making others sick by following the advice (63%) (Fig. 3D).

## Discussion

In this study, the vast majority of respondents reported that they had received sufficient information about the coronavirus. This information was disseminated through a variety of channels, both formal and informal. Press conferences from the government, health authorities’ websites and Norwegian news were reported to be the most relevant channels for all groups. Moreover, the majority of migrants reported high levels of trust in the Norwegian government and health authorities. Results were relatively similar among the five migrant groups. However, one difference that stood out was that the Polish group reported less trust than other groups in the effect of the recommendations on health and the Norwegian health authorities and government. All groups reported high levels of adherence to preventive measures but a perception that Norwegians do so to a lesser degree.

Concerning perception of health, the prevalence of self-reported suspected or confirmed COVID-19 cases was similar among all migrant groups. However, the reported level of COVID-19 infection risk varied among the groups, being twice as high for Tamils as compared to Somalis and Arabic speaking respondents. In addition, most migrants reported high self-perception of health, but this self-perception also varied among migrant groups. This variation is in line with the latest Norwegian study from SSB [18]. Somalis more often reported excellent or very good health, which concurs with the results of other studies in Norway [19, 20]. The higher COVID-19 infection risk and lower health levels reported by the Tamils could be explained by the fact that they are the oldest group among our respondents. These results correspond with the previous study on migrants performed in 2008 [18].

Our results on migrants receiving sufficient information are aligned with the results from a recent Finish report [21]. We are not aware of previous studies or reports on the different channels available for migrants to access health information. In this study, formal channels were considered more important than informal ones by all migrant groups, which might be surprising given that this information is in Norwegian. However, and even if we posed questions in the respondents’ mother tongues, the study population reported high levels of participation in the labor market and is, therefore, probably more integrated than other migrants in the same groups. Nonetheless, migrants with Somali background found informal channels to be more important than other groups did. This result can be explained by the strong oral culture in the Somali society [22]. In a context where much information about COVID-19 is being distributed via several channels, as reported by the respondents, gathering trustworthy information in migrants’ mother tongue in a specific channel would facilitate migrants’ access to information.

Research about migrants’ trust in public institutions in Europe is scarce. In a study including 26 European countries, Norway within them, Röder and Mühlau found that migrants had high levels of trust in host-country public institutions. Although health services were not evaluated in that study, trust in politicians was assessed [23, 24] and the results are consistent with the high level of trust in the Norwegian government obtained in our study. Results from the Polish group in our study are in line with the studies conducted in UK and Norway that showed that Polish migrants do not fully trust the host-country health services. These studies suggested the differences in the health system organization and treatment approaches they found in Norway compared to those in Poland as an explaining factor [25, 26]. Poles reported especially low levels of trust regarding how the government was handling the pandemic and was listening to their opinions. On the other side, trust among persons with Somali background in how the government has dealt with the pandemic was higher than that among all other groups. While there were few respondents from Somalia, a possible explanation may be that the government had a proactive campaign targeting this group at the time of the survey. Working migrants, a group in which Poles are overrepresented, were not specifically targeted. Qualitative research on these issues is needed to get a deeper understanding of the different answers.

As in our study, migrants in the referred Finish study also reported generally high adherence (over 90%) to most of the health preventive measures for COVID-19. These measures were similar to the one recommended in Norway at the time of our survey [21]. The high self-reported adherence and the perception that Norwegians followed recommendations to a lesser extent than our groups require more research and could be exaggerated to please the researcher. Different cultural perceptions of how strictly one should follow norms and to which degree one can trust his or her own group, as well as other social and cultural influences on behaviour, science communication, moral decision-making, leadership and stress and coping, have been lately proposed to understand self-report of adherence to COVID-19 recommendations [27, 28]. A further study should be performed to compare our results with Norwegians self-reported information on how they follow the rules.

### Study strengths and limitations

To our knowledge, this is the first study among migrants in Norway to study their access to information about COVID-19, trust in health authorities and adherence to recommendations. A strength in our study was that Inncovid. Norge is formed by researchers and health workers from the five migrant groups recruited. Knowing the communities and key persons in these environments facilitated the recruitment and achieving the targeted number of participants. However, the study has some limitations. First, the Somali speaking group is under-represented in our sample with only 33 respondents. This made it difficult to perform meaningful statistical comparisons among groups. Second, the results were obtained via a web-based questionnaire and although we used different channels and networks to reach the respondents within the five-selected migrant groups, selection bias may be present. Generally, respondents to our questionnaire were not representative of their populations, especially regarding their high employment status. They also differed in terms of gender and age, for which reason we weighted the results for the main outcomes. However, the different groups present characteristics as expected regarding length of stay and other variables. Thus, our results should be interpreted with caution for the generalizability of the findings to the migrant population in Norway. Third, given the survey nature of our study, we cannot disentangle the reasons behind our results, and further research is necessary to understand the differences in the outcomes we have identified among migrant groups. Last, in an ideal world, our results would be part of the NCP study, including representative samples of migrants, allowing the sound methodological comparison of results among groups and with the majority population, but this was not feasible at the time.

## Conclusion

Migrants report that they have enough information, but there are differences between groups in the most relevant channels used to get this information. Although most participants report a high level of adherence to recommendations, there is variation in levels of trust in the services and the authorities among the groups. Understanding how the different migrant groups are dealing with this pandemic is key to improve the dissemination of reliable and clear information and trust in the health authorities for the different migrant groups. This is essential to get long-term adherence to the recommendations in all segments of society.

## Data Availability

Yes, available upon request.

## Abbreviations

COVID-19: Coronavirus disease 2019
SSB: Statistics Norway
TSD: Service for Sensitive Data
NCP: Norwegian Citizen Panel
NOAS: Norwegian Organization for Asylum Seekers
